# The Costs of Preventing and Treating Chagas Disease in Colombia

**DOI:** 10.1371/journal.pntd.0000336

**Published:** 2008-11-18

**Authors:** Marianela Castillo-Riquelme, Felipe Guhl, Brenda Turriago, Nestor Pinto, Fernando Rosas, Mónica Flórez Martínez, Julia Fox-Rushby, Clive Davies, Diarmid Campbell-Lendrum

**Affiliations:** 1 Health Economic and Financing Programme, Department of Public Health and Policy, London School of Hygiene and Tropical Medicine, London, United Kingdom; 2 Centro de Investigaciones en Microbiología y Parasitología Tropical (CIMPAT), Facultad de Ciencias, Departamento de Ciencias Biológicas, Universidad de los Andes, Bogotá, Colombia; 3 Electrophysiology, Fundación Clínica Shaio, Bogotá, Colombia; 4 Centro de Investigaciones en Enfermedades Tropicales-CINTROP, Universidad Industrial de Santander-UIS, Guatiguará, Piedecuesta, Santander, Colombia; 5 Health Economics Research Group, Brunel University, Uxbridge, Middlesex, United Kingdom; 6 Disease Control and Vector Biology Unit, Department of Infectious and Tropical Diseases, London School of Hygiene and Tropical Medicine, London, United Kingdom; Universidad de Buenos Aires, Argentina

## Abstract

**Background:**

The objective of this study is to report the costs of Chagas disease in Colombia, in terms of vector disease control programmes and the costs of providing care to chronic Chagas disease patients with cardiomyopathy.

**Methods:**

Data were collected from Colombia in 2004. A retrospective review of costs for vector control programmes carried out in rural areas included 3,084 houses surveyed for infestation with triatomine bugs and 3,305 houses sprayed with insecticide. A total of 63 patient records from 3 different hospitals were selected for a retrospective review of resource use. Consensus methodology with local experts was used to estimate care seeking behaviour and to complement observed data on utilisation.

**Findings:**

The mean cost per house per entomological survey was $4.4 (in US$ of 2004), whereas the mean cost of spraying a house with insecticide was $27. The main cost driver of spraying was the price of the insecticide, which varied greatly. Treatment of a chronic Chagas disease patient costs between $46.4 and $7,981 per year in Colombia, depending on severity and the level of care used. Combining cost and utilisation estimates the expected cost of treatment per patient-year is $1,028, whereas lifetime costs averaged $11,619 per patient. Chronic Chagas disease patients have limited access to healthcare, with an estimated 22% of patients never seeking care.

**Conclusion:**

Chagas disease is a preventable condition that affects mostly poor populations living in rural areas. The mean costs of surveying houses for infestation and spraying infested houses were low in comparison to other studies and in line with treatment costs. Care seeking behaviour and the type of insurance affiliation seem to play a role in the facilities and type of care that patients use, thus raising concerns about equitable access to care. Preventing Chagas disease in Colombia would be cost-effective and could contribute to prevent inequalities in health and healthcare.

## Author Summary

Chagas disease is one of the most important vector-transmitted diseases in Latin America. Many patients with Chagas go undiagnosed for years, and because symptoms of the chronic condition are similar to those of other cardiac conditions, the burden of the disease is not evident. This leads to underestimation of the burden that Chagas disease places on healthcare resources, which in many cases translates into inadequate prioritisation for prevention. This study assessed the cost of Chagas disease in Colombia in comparison to the cost of prevention. Measuring the cost of treatment for Chagas disease is challenging. In this study we used a method that combines retrospective review of costs for prevention activities and for treatment of chronic Chagas disease patients, and we estimated utilisation of services using experts' consensus. We found that the cost of treating Chagas disease is substantial, even though many people are not receiving appropriate care. On the other hand, we show that preventing Chagas disease transmission through insecticide spraying activities is affordable in Colombia and therefore should be conducted more systematically. This study provides the basic inputs to conduct a full economic evaluation of Chagas disease prevention.

## Introduction

Chagas disease (American trypanosomiasis) has its origin in the American continent; however, due to increasing population migration to North America and Europe, the disease has expanded beyond its original borders. This parasitic disease has two principal routes of transmission to humans; by blood-sucking triatomine bug vectors and by blood transfusion. In 2004 it was estimated that 3 million Colombians (about 7% of the country's population) were at significant risk of infection as they lived in high-risk areas, and that 1.3 million were infected [1]. The main health impact of infection with Trypanosoma cruzi (Chagas) is chronic Chagas disease, which manifests in an estimated 10 to 30% of all infected patients [2]. This happens approximately 15 to 25 years after infection and consists of cardiomyopathies with or without congestive heart failure. For some patients the disease is fatal, while for others, expensive medical procedures such as implantation of pacemakers are needed.

The main prevention strategy to control Chagas infection is to reduce the triatomine vector population in areas of high endemicity, using insecticide residual spraying (IRS). Due to limited resources vector control programmes often target vector control by spraying (a) all houses in villages that are considered to be at highest risk, and (b) where risk is low, spraying only those houses with detectable bug infestations. Hence vector control programmes need to search for house infestation prior to IRS. The effectiveness and cost-effectiveness of IRS to control Chagas disease has been demonstrated by the Southern Cone Initiative [3] and in field trials [4]–[14]. These trials have also shown how results vary with, for example, choice of insecticide, dose and formulation, spraying strategy (proportion of houses sprayed, and degree of targeting), spraying methodology, timing and monitoring systems.

In Colombia, the departments with the highest infestation prevalences (percentage of houses with triatomines) are Arauca (21.1%), Casanare (10%), Santander (6.3%), Norte de Santander (5.2%), Boyacá (3.7%), Cundinamarca (1.9%) and Meta (1.7%) [3]. Vector control activities for Chagas disease in Colombia began in 1996 with a pilot vector control programme implemented in the Departments of Cundinamarca and Santander [15]. Prevention activities are not yet comprehensive or systematic, with both sampling and spraying activities varying significantly between the 32 Departments. This variation is partly due to competing priorities for limited funds, and uncertainty concerning which prevention strategies are most appropriate. Some Departments have implemented a strategy according to which all houses in the village are sprayed if house searches in a village detect a prevalence of ≥10%; otherwise only positive houses and those located within a 100 metre radius of the infested house are sprayed.

To date there have been no cost studies in Colombia to shed light on the resources needed to carry out systematic control activities. Likewise, there are no estimations of the costs borne by the health sector for treating Chagas disease patients. The few published studies available focus only on tertiary and specialised cardiology hospitals [16],[17]. Studies tend to ignore the level of utilisation of health care, assuming that all patients receive care and that this care is of a standard level [18],[19]. In reality, patients with chronic Chagas disease live mainly in rural areas, where access and quality of care is of a different quality compared to urban areas. Patients requiring specialised care may have to travel or move to urban areas (with their families), with potentially catastrophic effect on household income, or may simply fail to access such care.

This study provides the first estimates of the costs and utilisation patterns of Chagas patients in Colombia in three different levels of care. A gross estimate of the current burden of Chagas disease and the main input parameters required to explore cost-effectiveness analysis of Chagas disease prevention are also provided.

## Methods

### Costing vector control activities

Three departments (Norte de Santander, Santander and Boyacá) from the most endemic regions of Colombia participated in the study. In a retrospective analysis, a total of 6389 houses that registered vector control activities in the last 6 months was included, 3084 houses for entomological survey and 3305 for spraying. The specific municipalities and villages that compose the sample (see Table 1) had been prioritised for vector control activities by the Chagas control programme managers the previous year. All localities in which interventions were made were in rural areas; however, the dispersion of houses varied across localities. We did not conduct household surveys to carry out this analysis; records on resources used in these activities were provided by the staff in charge of vector control programmes, who were also interviewed about the operational aspects of these activities.

**Table 1:** Unit costs per house surveyed and sprayed (US$, 2004).

Cost item/ Department (Municipality)	Norte de Santander (Cucutá)		Santander (Macaravita, Capitanejo)		Boyacá (Soatá)		Boyacá (Moniquirá)		Total weighted average	
***Cost per house surveyed***										
***Number of houses***	***43***		***285***		***795***		***1961***		***3084***	
***Cost categories***										
Staff salaries	216.8	46%	732.1	33%	1664.0	54%	4181.1	54%	6794.0	50%
Per diems	164.9	35%	759.0	34%	1038.6	33%	2614.0	34%	4576.6	34%
Supplies	14.1	3%	87.9	4%	163.7	5%	402.9	5%	668.6	5%
Transport		-	560.8	25%	163.1	5%	392.5	5%	1116.4	8%
Indirect costs/ overheads	75.4	16%	69.0	3%	71.2	2%	135.8	2%	351.4	3%
***Total Costs***	***471.3***	***100%***	***2208.8***	***100%***	***3100.5***	***100%***	***7726.3***	***100%***	***13,506.9***	***100%***
***Unit cost per house***	***11.0***		***7.8***		***3.9***		***3.9***		***4.4***	
***Cost per house sprayed***										
***Number of houses***	***61***		***661***		***761***		***1822***		***3305***	
***Cost categories***										
Staff salaries	397.2	15%	2927.7	27%	4527.7	20%	10 656.5	20%	18,509.18	21%
Per diems	294.8	11%	3035.5	28%	2776.8	12%	6548.1	12%	12,655.25	14%
Insecticide (Deltamethrin)	1740.9	67%	2343.7	22%	13 759.7	61%	33 133.0	62%	50,977.32	57%
Transport	79.0	3%	560.7	5%	441.7	2%	1014.2	2%	2,095.54	2%
Other direct supplies	48.9	2%	376.2	4%	186.7	1%	438.0	1%	1,049.78	1%
Indirect costs/ Overheads	23.8	1%	1497.4	14%	787.4	4%	1740.5	3%	4,049.04	5%
***Total Costs***	***2,584.6***	***100%***	***10,741.3***	***100%***	***22,479.9***	***100%***	***53,530.4***	***100%***	***89,336.1***	***100%***
***Unit cost per house***	***42.4***		***16.3***		***29.5***		***29.4***		***27.0***	

### Entomological surveys

In all sites the entomological surveys consisted of timed manual collections (one –person-hour per house), combined with the distribution of pots to householders to collect triatomines after the search. The entomological surveys also included geographical reconnaissance (for mapping in *Geographical Information Systems*), epidemiological stratification and the implementation of *Triatomine Information Posts* (TIP) in the villages. In these posts, usually based in public schools, neighbors can obtain information and report triatomines found in houses. TIPs constitute the basis of post-spraying surveillance.

The surveys were conducted by trained assistants and technicians in team sizes that varied depending on the area of intervention. For example, in the field sites in Boyaca, teams consisted of 3–6 people, and averaged 10 houses surveyed per person-day. In Santander and Norte de Santander, with more remote rural areas, dispersed houses, and poor or absent roads, the rate of surveying was about half this value.

### Spraying activities

In Santander and Norte de Santander spraying teams comprised one technician or supervisor and 3 or 4 assistants. In Boyacá spraying teams comprised 5 assistants and a driver. Technicians in Boyacá participated only in the organisational aspects of spraying, which included conducting a sensitisation campaign using Information Education and Communication (IEC) strategies, and training assistants in spraying techniques. These also engaged key community stakeholders, delivered leaflets and organised community workshops in the villages. In these workshops, households were taught to reorganise their house contents and their peridomicile so as to reduce the risk of triatomine reinfestation after spraying.

Deltamethrin (K-Othrine SC 50 Bayer) was the main insecticide used in all sites; however, in Norte de Santander Lambdacyhalothrin (Icon 10 PM) was also used to spray some houses. A litre of insecticide was used to spray approximately 10 houses. Follow-up and quality control of spraying were carried out by supervisors in 10% of the houses intervened in Boyacá. This was not done in Santander or Norte de Santander.

Salary rates of field workers (i.e. full cost of employment per month in US$ of 2004) were slightly higher in Norte de Santander and Santander, where a technician and an assistant earned $616 and $541, respectively. These were lower in Boyacá where a technician earned $546 and an assistant $418. However, in Boyacá salaries of supervisors were around $1,180 per month (compared to $760 in Norte de Santander). Per diems were higher in Santander, with a range from $15 to $25 per person-day, and lower in Boyacá with rates between $10 and $15 per person-day.

### Costing methodology for vector control

From a healthcare provider perspective, the cost per house entomologically surveyed and the cost per house sprayed were determined using standard costing methodology [20],[21] We combined an *ingredients* approach (i.e. aggregating one by one the resources involved in each activity) with *step-down* method (i.e. identifying total monthly/annual expenditures per category and disaggregating them for our activities/time of interest) [21]. We differentiated between direct and indirect (or overhead) costs. All cost data were adjusted for inflation to 2004 prices and converted to 2004 US dollars. The exchange rate observed in December 2004 was 2,430 Colombian pesos (COP) to one US$, as reported at www.oanda.com. Direct costs included field workers (their salaries and per-diems), supplies (consumables used for entomological surveys and for spraying) and transport (use of vehicles, fuel and maintenance costs). Straight line depreciation was used to reflect the cost for the use of capital items, mainly vehicles (10 years lifespan) and Hudson X-pert type hand-compression sprayers (5 years). Indirect costs (or overheads) in all sites included professionals time (their salaries and per-diems) and transport costs associated with planning and supervision of control activities. These covered personnel from the department and municipalities.

In the case of Boyacá, direct costs also included the sensitisation campaigns, IEC strategies, and the community workshops, as these activities were carried out systematically in all villages before spraying. These costs were based on salary rates, per-diems and transport of the personnel from the municipality that carried out these activities. For simplicity, we did not include as indirect costs the time of community stakeholders and households that participated in these activities. Thesewere not paid for their participation on these activities

### Costing treatment of chronic Chagas disease

Treatment costs were evaluated from a payer perspective (i.e. patient-insurance-government) in line with new (insurance-based) financing system. We differentiated between 3 levels of care: *basic* (primary care where Chagas disease is not diagnosed and suspected cardiomyopathy patients are referred on); *intermediate* (district and regional hospitals where diagnosis is possible along with non-surgical or palliative and preventive care for cardiomyopathy), and *specialised* (metropolitan hospitals that can carry out complex procedures such as pacemaker implantation).

A total of 63 patients' records was included in the analysis. Of these, 31 were identified in the municipal hospitals of Soatá and Moniquirá (Department of Boyacá), where vector control activities were evaluated. Each of these hospitals serves a population of around 20,000 inhabitants. The other 32 patients were selected in the specialised cardiovascular hospital in Bogotá, *Fundación Clínica Shaio*. The protocol or consultation of patients' records was approved by the committee of ethics and research of the *Fundación Clínica Shaio*. Based on this approval, the directors of the three institutions gave permission for consultation of patients' records. These institutions were selected to represent a relevant mix of care, with Boyacá hospitals (located in semi-rural settings) classified as providing an *intermediate* level of care, while the Fundación Clínica Shaio provides *specialised* cardiovascular care. Previous links on Chagas disease research facilitated access to these institutions. We did not include primary healthcare centers in the sampling as patients with symptoms of cardiomyiopathy are referred on, normally without Chagas disease diagnosis. However, we still estimated cost associated with this level of care (see next section).

### Data collection and data analysis on treatment costs

In the three hospitals, records of all patients registering visits due to heart disease between January 2000 and August 2004 were reviewed. Patients were included in the study if (1) Trypanosoma cruzi infection was confirmed, and (2) the presence of Chagas cardiomyopathy symptoms was the reason for seeking care. At the basic level of care, costs of treatment were drawn from the estimations on utilisation carried out by the expert panel and the unit costs of consultations. The former, included the frequency of visits to a General Practitioner (GP) and the probability of hospitalisation per year.

For each hospital a database was created in Excel to register and analyse chronologically all services that each patient had received since the first chronic Chagas-related visit. The duration of treatment varies from patient to patient; while some patients have completed one year, others have been on treatment for up to ten years. The services consumed by the patients were grouped into ambulatory care, visits to emergency services, in-patient bed-days, clinical and other diagnostic investigations or procedures, surgical procedures, and other medical procedures (e.g. rehabilitation if applicable). Total costs were obtained by multiplying unit costs (based on hospital charges updated to 2004 values) by the quantity of services consumed. For valid interpretation, results are expressed as the weighted mean cost per patient per year for each level of care.

In each level of care, costs were analysed separately for patients with Cardiomyopathy (C) with congestive heart failure (CHF) and patients with C without CHF. Given the relatively small sample size and the focus of the study on economic burden, we did not seek to associate specific levels of disease progression with costs. However, for the specialised level of care, a detailed account of the interventions and procedures received by patients is provided in the results section.

### Care-seeking behaviour and utilisation patterns

Consensus development techniques, such as Delphi methods, use expert panels to gather information for which there is not strong evidence. These have been used in a variety of contexts [22],[23] including economic evaluations [24]. As the proportion of patients that access each level of care had not previously been measured, nor was it known how soon patients seek care after onset of first symptoms of cardiomyopathy, a panel of experts from Colombia was assembled. The panel included medical doctors (GPs, cardiologists), Chagas vector control managers and other professionals related to health care arrangements in general and Chagas disease in particular. All participants were sent instructions with a set of 9 forms for completion in advance of the panel meeting. These aim to specify healthcare facilities that were likely to provide services to Chagas chronic patients and to identify pathways of care. Specifically, participants were asked: (1) to classify these facilities to one of the three levels of care; (2) to describe the referral system between the three levels of care with focus on the empirical rather than the written norms; (3) to estimate healthcare seeking behaviour at first symptoms of Chagas chronic disease (what proportion seek care and where); and (4) to estimate dynamics of the pathway of patients through the healthcare network after first symptoms.

The Delphi exercise was conducted in 3 steps; input, discussion and review (iterative in each round) and conclusions:

- **Input:** All available and recent data (from local sources) on various determinants of utilisation in Chagas disease were reviewed, such as epidemiology, demographic and healthcare structure. Healthcare insurance coverage rates (urban/rural), care-seeking and utilisation patterns were reviewed from the results of a national household survey [25]. Further information on the current situation of treatment of chronic Chagas disease in line with the new financing system was reported by representatives of the central level [26].
- **Discussion and review:** Participants were separated into three groups, each with a range of specialization and geographical origin of participants. In the first round of consensus, participants were instructed to generate new “reviewed” individual estimates (after presentation of all available evidence). A second round included discussion among participants of each group, after which consensus was reached to produce only one set of estimates per group.
- **Conclusion:** Finally the three groups presented their consensus results, followed by, discussion, especially about parameter estimates that varied widely across groups. After this final discussion, groups had a chance to change any estimated parameter values. The final output of the Delphi was estimated by averaging results from the three groups. The parameter estimates generated through this process included:
  - The proportion of patients that do not seek care (apart from palliative care near death).
  - The proportions that seek care at each level.
  - The average number of years that patients are treated in each level of care.
  - The proportion of patients with C, with and without CHF, that seeks each of the three levels of care.

Using the information on unit treatment costs and the parameters on utilisation derived from the consensus exercise, we estimated the expected lifetime costs of treatment for a patient with chronic Chagas disease in Colombia. We used a 3% discount rate on future costs to reflect the current long-term opportunity cost of capital in Colombia.

## Results

### Vector control activities

Total and average costs are presented in Table 1. The average weighted cost per house surveyed was found to be $4.4, while the average cost per house sprayed was $27. For surveying, on average, salaries accounted for 50% of the total cost, followed by per-diems (34%) and transport (8%). However, variation can be observed across sites, especially with respect to salaries, transport and indirect costs. The highest mean cost is registered for *Norte de Santander*, where the intervention area had no access for vehicles and the houses were geographically dispersed.

In relation to spraying activities, the cost of the insecticide was on average 57% of the total cost per house sprayed. However, in *Santander* it was only 22%, as the insecticide had been purchased through the central government at the comparatively low price of US $33 per litre. In Colombia the central level negotiates the purchase of high volumes of insecticide for malaria and Chagas disease programmes, based on the Departments estimation of needs budgeted in the previous year. When Departments run out of centrally-purchased insecticide, they have to purchase it directly from private providers. This was the case of *Norte de Santander* and *Boyacá* where US $180 per litre was paid. Such disparities have been described in a recent paper on the cost of spraying for malaria control [27]. The next most important resource inputs in the cost per house sprayed were salaries at 21%, followed by per-diems at 14%.

Treatment costs at each level of care are summarised in Table 2; these costs are disaggregated further in the subsequent tables. Average annual costs of chronic Chagas disease patients with C with CHF were higher than costs for patients with C without CHF in all levels of care. The costs of care rise considerably from primary to tertiary care providers. In the *basic level of care* annual treatment for patients with C with CHF consists of 3 GP visits and 0.58 days of hospitalisation, while for patients with C without CHF this consists of 5.3 GP visits and 0.25 days of hospitalisation.

**Table 2:** Annual expected cost of patients in different level of care (US$, 2004).

*Mean cost per patient-year*	*Cardiomyopathy with CHF*	*Cardiomyopathy without CHF*
**Basic level of care**		
Average cost	$51.4	$46.4
**Intermediate level of care (ILC)**		
H. Regional de Soatá	$237.9 (n = 11)	$188.0 (n = 5)
H. Regional de Moniquirá	$275.0 (n = 15)	
**Weighted average (ILC)**	**$259.3 (n = 26)**	**$188.0 (n = 5)**
**Higher level of care**		
Fundacion Clínica Shaio	$7,980.9 (n = 17)	$3,651.5 (n = 15)

n = number of patients in the analysis.

### Treatment results for intermediate level of care

Patients from Soatá Hospital have a mean age of 68.3 years (range 49–85). More than half of them (56%) were from Soatá municipality while the rest were from neighbouring municipalities, and 63% were males. In Moniquirá Hospital the age structure was very similar: mean age 67.2 years (range 49–83). Here only one patient did not come from Moniquirá municipality, and 47% were males. We did not collect data on patients' addresses and therefore we cannot state with precision the proportion of patients that lived in rural areas. However, it is highly likely that most of the patients seeking care in Soatá and Moniquirá hospitals (both located in semi-rural areas) were infected and possibly still live in rural areas.

All patients selected in Moniquirá hospital had C with CHF. In these two hospitals 4 patients had died due to the disease. All patients sampled in this level of care were insured, with 93.5% belonging to the *subsidised* healthcare regime, meaning that these patients were relatively poor with an income under one *legal minimal salary* (≤US $ 138 per month). The remaining 6.5% were in the *contributive* regime.

Table 3 shows that in the intermediate level of care, patients with C with CHF experienced at least twice as many hospitalisation days as patients with C without CHF. However, the latter group had more GP visits per year. Thus, while hospitalisation is the larger cost component for patients with C with CHF, medicines are the larger component for patients with C without CHF. Patients from the hospital of Moniquirá had a longer average time on treatment at the time of research (7.5 years), while patients form Soatá hospital have been on treatment for just over 2 years on average.

**Table 3:** Total cost per patient-year and services consumed in intermediate level of care (US$, 2004).

Mean cost/services per patient-year	H. Soatá (C with CHF)	H. Moniquirá (C with CHF)	Cost profile (C with CHF – both hospitals)	H. Soatá (C without CHF)	Cost profile (C without CHF)
***Cost categories***	n = 11	n = 15		n = 5	
Outpatient visits	20.4	15.2	7%	33.7	18%
Diagnostic investigations	40.5	26.9	13%	28.6	15%
Medicines	66.4	93.3	31%	78.4	42%
Hospitalisation	110.7	139.6	49%	47.3	25%
***Total cost (US$)***	***237.9***	***275.0***	***100%***	***188.0***	***100%***
***Years on treatment***	2.6 (1.0–13.8)	7.5 (1.0–22)		2.3(1.0–4.4)	
***Mean number of health services consumed per year***					
**Outpatient visits**					
GP visits	2.2	1.6		4.5	
Visit to specialist	0.4	0.3		0.4	
Emergency admissions	0.5	0.3		0.3	
**Diagnostic tests**					
Electrocardiograms	1.1	0.7		1.0	
Various laboratory tests	3.5	1.4		2.2	
Thorax x-ray	0.6	0.4		0.3	
**Hospitalisations**					
Bed-days	2.4	2.6		1.0	
Other procedures (oxygen administration, respiratory therapy, etc)	-	2.3		-	

n = number of patients in the analysis.

### Treatment cost in the specialised level of care

In the *Foundación Clínica Shaio* 75% of the 32 patients were females. Patients were generally younger than those from intermediate care hospitals, with a mean age of 53.4 years (range 34–78); 38% of the patients came from Boyacá Department (the nearest Chagas disease endemic Department), 25% from Santander and 37% from other endemic departments. There were 17 patients classified as C with CHF, 13 classified as C without CHF, and the type of cardiomyopathy remained unclear in two patients. One patient had died due to the disease. All patients were insured through the contributory regime, and therefore were less poor than those who used intermediate level care.

The services that patients received in the specialised level of care increased considerably in complexity and costs. Table 4 shows that the largest component of cost was surgical procedures, reaching 41% and 55% of total annual costs for C with and without CHF, respectively. Overall, there were 16 patients that had a pacemaker implanted (9 with C with CHF and 7 without). Two other patients had cardiac transplants, one had a cardio-defibrillator implant, and another had a cardiac re-synchroniser. Medicines and drugs were the second highest component of cost, reaching 24% and 10% for patients with C with and without CHF, respectively. The patients had been under treatment for a relatively short time, on average 1.4 years (range 1–9). In this level of care, patients with C with CHF received considerably more services than those without CHF (see Table 4). For example, the former averaged 20 attendances by specialists per year, compared to only 8 for patients with C without CHF. On average, a patient with C with CHF had 12.6 days of general hospitalisation and 1.6 days in an *intensive care unit* (ICU) per year. This is reduced to 5.8 days of general hospitalisation and 1 day in ICU for patients with C without CHF. Finally, the number of laboratory investigations was three times more in patients with C with CHF.

**Table 4:** Distribution of mean cost by type of service and cardiomyopathy in specialised level of care (US$, 2004).

*Mean costs per patient-year*	C with CHF		C without CHF	
	n = 17		n = 13	
Ambulatory care (includes outpatient visits and telemetry)	48.5	1%	42.3	1%
Hospitalisation				
Emergency admissions	11.8	0%	3.4	0%
Bed-days general	504.1	6%	190.5	5%
Bed-days in intensive care unit (ICU)	504.2	6%	269.7	7%
Procedures in hospitalisation				
Electrocardiography	58.1	0%	21.2	0%
X-rays	80.6	1%	29.8	1%
Laboratory	416.4	5%	72.9	2%
Medicines & drugs	1,887.8	24%	372.1	10%
Surgical procedures & surgical elements	3,261.8	41%	2,018.3	55%
Electrophysiology	319.4	4%	241.3	7%
Rehabilitation, physiotherapy, etc.	543.3	7%	256.9	7%
Other procedures	344.9	4%	133.1	4%
***Total annual cost per patient***	***7,980.9***	***100%***	***3,651.5***	***100%***
***Mean services per patient-year***				
Outpatient appointments	1.3		0.6	
Hospital procedures				
Emergency admissions	0.3		0.2	
Attendances by specialist	20.0		8.1	
Bed-days general	12.6		5.8	
Bed-days intensive care unit	1.6		1.0	
Laboratory investigations	68.6		21.4	
Thorax x-ray	2.9		1.5	
Cardiology diagnostic techniques	1.1		0.9	
Electrophysiological assessments	0.4		0.5	
Imaging heart study	0.7		0.1	
TAC (computed-tomography)	0.1		0.1	
Surgical procedures				
Pacemaker implantation	0.4		0.4	
Cardio-defibrillator implantation	0.02		-	
Cardiac re-synchronizer	0.02		-	

n = number of patients in the analysis.

### Health seeking behaviour and healthcare utilisation

**Figure 1:**
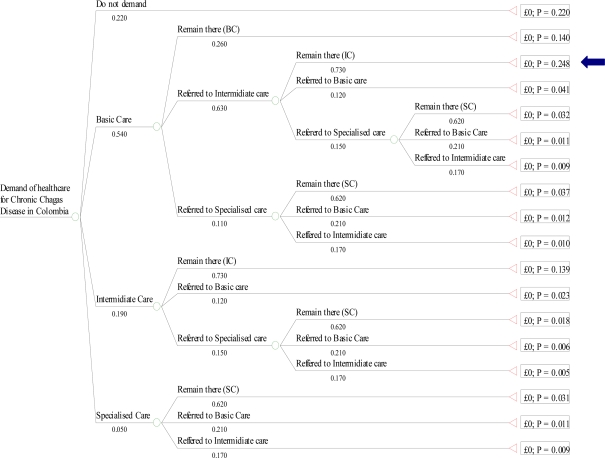
Health seeking behaviour and pathway of Chagas disease patients through the levels of care. This decision tree shows all possible routes that a patient can follow to be finally treated predominantly in one level of care. The letter “p” in the box to the right indicates the probability that a patient follows that specific route of care. For example, the tree arm whose terminal node is next to the arrow corresponds to the route where the patient seeks care first in a facility that provides basic care, s/he is then referred to a facility that provides intermediate level of care and will remain there during the course of the treatment. This pathway of care will be followed by 24.8% of the patients (p = 0.248).

The consensus of experts was that the majority (54%) of patients feeling the first symptoms of chronic Chagas disease will seek care first in the basic level of care, 22% will not seek care, 19% will go straight to an intermediate level of care (such as the Hospital of Soatá or Moniquirá) and only 5% will go immediately to a specialised level of care (see these values on the left of each arm in Figure 1). Patients tend to seek care late, when the symptoms worsen, which occurs on average ten years after clinical onset of the cardiomyopathy. When patients seek care they follow different pathways within the health care system depending on various factor, such as insurance coverage, severity of the condition, place of residence and patient's resources. They are eventually treated predominantly in one specific level of care. For simplicity we did not attempt to model annual transition probabilities across levels of care, instead we used a decision tree to present the Delphi consensus estimates (Figure 1). These estimates were used to ascertain average lifetime utilisation patterns for patients with chronic Chagas cardiomyopathy in Colombia.

Figure 1 shows all possible routes that a patient can follow when seeking treatment to be finally treated predominantly in one level of care. To ascertain the total proportion of patients that are treated in each level of care, we sum up for each level of care, the probability estimates appearing in the terminal node of the arms that end in that specific level of care. Thus, derived from Figure 1, 24% of the patients are treated predominantly in facilities corresponding to basic level of care, while the majority (42%) is predominantly treated in intermediate level of care and only 12% are treated in the specialised level of care. The proportion that does not seek care, apart from palliative care near death, was estimated at 22%. For costing purposes, it was assumed that seeking care near death would cost, on average, the equivalent of two years of intermediate level care.

Merging information on annual cost of treatment (Table 2) and utilisation patterns (Figure 1) we estimated an average expected annual cost per chronic Chagas patient in Colombia of $ 1,028 as shown in Table 5. This figure accounts for the probability that patients seek care in different levels of care according to type of cardiomyopathy and patient characteristics. Lifetime duration of chronic cardiomyopathy (in years) was also drawn from the expert panel for the different groups of patients. This allowed the estimation of the approximate lifetime costs on treatment for a chronic Chagas disease patient in Colombia. Table 5 shows that, on average, a chronic patient in Colombia would cost $11,619 (US$, 2004) in treatment during their lifetime.

**Table 5:** Total expected mean cost per patient, weighted by care seeking and utilisation patterns (US$, 2004).

Patient group	Annual treatment cost	Utilisation rate by level of care a	CHF & NCHF split by level a	Disease duration years a	Estimated lifetime cost b
Do not seek care CHF	34.9 c	0.22	50%	20	$518.6
Do not seek care NCHF	19.2 c	0.22	50%	30	$376.0
Basic level of care CHF	51.4	0.24	80%	30	$569.0
Basic level of care NCHF	46.4	0.24	20%	40	$676.7
Intermediate level of care CHF	259.3	0.42	85%	30	$2,870.5
Intermediate level of care NCHF	188.0	0.42	15%	40	$2,741.9
Higher level of care CHF	7,980.9	0.12	90%	30	$88,350.5
Higher level of care NCHF	3,651.5	0.12	10%	40	$53,255.6
***Mean expected cost per patient***	***$1,028.21***				***$11,618.57***

a All these estimations were obtained from the consensuses of the expert panel (refers to figure 1).

b Lifetime costs were calculated as the present value of annual costs accounting for the disease duration (minus ten-year delay for seeking care) and 3% discount rate.

c Patients who do not seek treatment care, apart from palliative care near death, were assumed to spend an equivalent cost to two years in intermediate level of care (value also annualised).

## Discussion

The cost results of this study and those reported by the international literature are shown in Table 6. The costs of vector control activities were lower in comparison to previous studies and these were highly dependant on the cost of the insecticide. Treatment costs varied widely among different levels of care and between patients with C with and without CHF. Care seeking behaviour and utilisation patterns suggest that patients delay treatment; most of them received care of an intermediate level (42%); few would use specialised levels of care (12%), and 22% would remain untreated except for palliative care near death.

**Table 6:** Data on cost of prevention and treatment of Chagas disease presented by other (selected) studies.

*Study*	*Setting, period*	*Cost reported*	*US$ of the respective year (range)*
**Vector control activities**			
This Study	Colombia, 2004	Cost per house surveyed	$4.4 (3.90–10.96)
	Departments: Santander, Norte de Santander and Boyacá	Cost per house sprayed	$27. (16.25–42.37)
Oliveira-Filho (1989) [7]	Municipality of Posse, State of Goiás, Brasil	Cost per house sprayed per year protected	$20.1–29.4 Varies for different insecticides and formulations
Basombrio & colleagues (1998) [28]	Argentina Department of Anta, Province of Salta, Argentina	Cost per house sprayed	$41.04 Concentrated settlements,
			$64.98 Disperse settlements,
Kroeger & colleagues (2002) [27]	Colombia, 2002	Cost of house sprayed for malaria vector control	$36.8 In nearby communities
			$47.8 In distant communities
Chuit & colleagues (1992) [33]	Argentina, County of Rio Hondo in Santiago del Estero Province.	Two methods of house surveillance for triatomine infestation were tested: conventional method (i.e. direct search by one person-hour per house) and sensor boxes	$17 house-year conventional method
	Years: 1985,-1987		$3.4 house-year sensor boxes
			$60 cost per house sprayed
**Cost of treatment**			
This Study	Colombia, 2004	Annual and lifetime cost of treatment for cardiomyopathy adjusted by utilisation and care seeking behaviour	$1028 (46.4–$7,980.9)
	Hospitals in Department of Boyacá and on in the Metropolitan city of Bogotá		$11,618.57 ($569.0–$88,350.5)
Akhavan (1996) [19]	Brazil,1996 (nationwide estimation)	Lifetime cost of treatment, including patients in the indeterminate stage of the disease	$1,140–$55,159 (range)
Schenone (1998) [17]	Chile, public sector hospitals in endemic areas, 1998	Cost of chronic disease treatment per patient-year	$439–584 (range)
Basombrio & colleagues (1998) [28]	Argentina Department of Anta, Province of Salta, Argentina, 1998	Annual costs of treatment for chronic heart disease	$603.62 chronic heart disease
Vallejo & colleagues. (2002) [16]	Mexico (National Inst. of Cardiology)	Cost of chronic disease treatment, per admission	$4,463–$9,601 (range) normal referral
			$6,701–$11,839 (range) emergency admission

The main cost component of the vector control programme is the cost of spraying houses. Our finding of $27 per house seems very favorable when compared to previous cost estimations e.g. for the southern cone [28]. This cost was also lower than the cost of spraying houses for malaria control in Colombia, where using mainly Lambdacyhalothrin [Icon 10WP] a high level of insecticide wastage per house was reported [27]. In this study, insecticide accounted for 57% of total costs. However, most of the sites had paid a relatively high price, as purchases have been made individually from the Department rather than from the Ministry of Health (MOH). In 2004 the MOH's national negotiated price was only $33 per litre for Deltatmethrin (K-Othrine sc 50 Bayer). This suggests that better planning and coordination would lead to significant savings in the costs of spraying houses. We estimated that if the three Departments would have used insecticide bought at central level prices, the mean cost per house sprayed could have been reduced from $27 to $16.

Annual treatment costs for chronic Chagas disease patients varied from $46 for a patient with C without CHF treated in *basic care* to around $7,900 for a patient with C with CHF that used the *specialised level* of care. This 30-fold increase in costs reflects the different type of care (and potential differences in health outcomes) offered in these institutions. However, this dichotomous situation observed between intermediate and specialised care cannot reliably be generalised countrywide, as it is possible that other hospitals in Colombia, such as provincial hospitals, offer services whose complexity and costs lie between these two types of facilities. Among the international literature reporting Chagas disease patients' treatment costs, Schenone [17] reported annual costs from public hospitals in Chile. These costs lie between our intermediate care costs and those for specialised levels of care, suggesting that the care provided in Chile was potentially more complex than that provided in the municipal hospitals of Colombia but not as complex as this offered by the Fundación Clínica Shaio. The costs observed in the *Fundación Clínica Shaio* are in line with a Mexican study from the *National Institute of Cardiology* where the cost per patient admitted varied from $4,463 to $11,839 depending on the type of admission [16]. Estimations done in Brasil [19], reported lifetime costs of an infected patient (irrespective of developing chronic condition) averaging $3,864, which as expected, is lower than our estimation of $11,618.57 which considers only chronic patients.

Many of the previous studies have not reported or reflected on patients' care-seeking behaviour and utilisation patterns. The expert panel estimated that 22% of patients do not seek care and from those who do so, not all of them have access to a specialised level of care such as a cardiology hospital. In Colombia, the healthcare financing system is undergoing a major reform that seeks to improve access to health care by reaching universal coverage [29]. However, data for the time of the study showed that only 52% of the population had insurance coverage, and in rural areas this percentage dropped to 40%. Bitrán and colleagues have reported that uninsured patients restrict their demand for healthcare even when they seem to have more needs [30]. Although in theory uninsured patients have the right to receive care, there are concerns as to the extent that this materialises in practice. The health insurance system implies that different third-party-payers (or insurers) reimburse health facilities that serve *subsidised*, *contributive* and *uninsured* patients. It has been reported that the insurers that cover the *uninsured* and the *subsided* patients have faced financial solvency problems thus delaying reimbursements to healthcare facilities. Hospitals on the other hand, which have been made autonomous in this reform and therefore seek to increase revenues, avoid the admission of patients whose insurers do not keep payments up-to-date, and in some cases have implemented up-front out-of-pocket payments of drugs and other procedures for these patients, which are often poorer than average [26].

We acknowledge the limitations of our study in terms of the relatively small sample of patients (64), as well as the potential limitations of the Delphi exercise at ascertaining care seeking and utilisation patterns. Nonetheless, we believe that this information can help to guide policy decisions on prevention of Chagas disease. Vector control managers in Colombia (as well as in other countries) can use these costs and methods as a reference when planning future control activities. As the insecticide is the main cost driver of spraying, they need to plan in advance, and aim to purchase most or all insecticides at the lower prices that can be achieved from the central level. We found that different sites use different operational arrangements when carrying out vector control activities. Vector control managers need to use resources wisely, engaging in multi-sectorial approaches and involving community stakeholders, as these can play an important role on activities such as surveillance. Education and community participation can have an important impact on the sustainability of vector control, both for Chagas disease and other infectious diseases. The impact of community participation in the sustainability of vector control activities for Chagas disease has been recently demonstrated by Gürtler and colleagues [31] in the Gran Chaco, Argentina.

An area that deserves further research is the degree of (equitable) access to a standard level of care for chronic Chagas disease patients in Colombia. In our study, patients who used intermediate levels of care were poorer compared to those that used specialised care. In the former group 93% were members of the *subsidised regime* (i.e., they are entitled to a much more limited package of care than those in the *contributive regime*) while patients that used specialised care were all members of the *contributive regime*. Overall, we did not found uninsured patients demanding care for Chagas cardiomyopathy, despite the fact that 60% of the population in rural areas were uninsured in Colombia in the year 2000 [30]. This poses questions as to how the lack of insurance coverage could deter care seeking, not only in relation to Chagas disease patients, but even more generally.

The extent to which the costs of vector control activities can be offset by potential savings on treatment averted is a more complex analysis that lies outside the scope of this article. Chagas disease has specific characteristics, because not all patients develop chronic disease, and for those affected, this happens 15–20 years after infection. Current control activities will deliver benefits mainly in the future, as fewer people become infected and develop the chronic condition. Thus, applying the common practice in economic evaluation of adjusting costs and health outcomes for time difference, process known as *discounting* [21], would have the impact of reducing the benefits of Chagas prevention in comparison to control of other diseases, for which benefits would accrue sooner.

In general, vector-borne transmitted diseases require the use of modelling to evaluate control activities in conjunction with infections averted. Thus, an estimation of current burden of Chagas disease in Colombia is challenging, as it is not known how many individuals are currently chronic patients and whether their seeking behaviour is well represented by the estimate done by the expert panel. However, using estimations of 1.3 million infected Colombians [1], we can estimate the approximate annual cost of treatment across the population. If 20% develop chronic conditions, this would translate into 260 thousand people receiving care, with annual costs of treatment of $1,028 per person, leading to an economic burden of around US$ 267 million per year (US$, 2004). This estimation does not include infected patients who have not developed chronic conditions. The latter needs to be investigated to ascertain the extent to which these patients are also a burden on the healthcare system. According to a national entomological survey, there were 178,519 dwellings considered of high risk for Chagas disease in Colombia [1], which should be prioritised for insecticide spraying. Using our results on costs of prevention, spraying these houses would cost nearly five million dollars, which is around 2% of expected annual expenditure on care. Again, these figures need to be treated with caution since they are not directly comparable, as it may be appropriate to apply discounting to the future benefits of vector control. Furthermore, reinfestation, implies that a long term surveillance system to survey and re-spray reinfested houses, needs to be in place even after spraying all the houses. These rough estimations nonetheless seem to suggest that preventing Chagas disease in Colombia may be cost-effective in comparison to treating disease, with the potential of accruing not only economic savings, as well as more importantly, avoiding disability and suffering. A more comprehensive analysis on the cost-effectiveness of control activities over time is addressed in a separate paper [32], where the incremental net benefit of control activities in specific geographic locations of Colombia is determined.

## Supporting Information

Alternative Language Abstract S1 Translation of the Abstract into Spanish by Felipe Guhl
(15 KB PDF)
