# Supplementary material for: The Costs of Preventing and Treating Chagas Disease in Colombia
Source: PLoS Negl Trop Dis. 2008 Nov 18;2(11):e336. doi: 10.1371/journal.pntd.0000336 (PMC2581604; doi:10.1371/journal.pntd.0000336)
Supplement: Alternative Language Abstract S1 — Translation of the Abstract into Spanish by Felipe Guhl (15 KB PDF) [file pntd.0000336.s001.pdf]

Los costos de prevención y tratamiento de la enfermedad de Chagas en Colombia

Resumen

Objetivo: Reportar los costos de la enfermedad de Chagas en Colombia, en relación a los programas de control vectorial y los costos de atención a los pacientes con cardiopatía chagásica crónica.

Métodos: Los datos fueron colectados en Colombia en 2004. Se llevó a cabo una revisión retrospectiva de costos de programas de control vectorial incurridos en áreas rurales. Se incluyeron 3,084 viviendas encuestadas para evaluar infestación por insectos triatominos y 3,305 viviendas rociadas con insecticida. Un total de 63 historias clínicas de tres hospitales diferentes fueron seleccionadas para realizar una revisión retrospectiva de los recursos utilizados. Se aplicó una metodología con la participación de expertos locales para estimar el comportamiento de búsqueda de servicios de atención médica por parte de los pacientes y así complementar los datos observados de utilización.

Hallazgos: El costo promedio por encuesta entomológica por casa fue de $4,4 (en dólares de EEUU de 2004), mientras que el costo promedio de rociar una vivienda fue de $27. El mayor costo derivado del rociamiento fue el precio del insecticida, el cual varió significativamente. El costo de un paciente chagásico crónico en Colombia varió entre $46,4 y $7,981 por año, dependiendo de la severidad y del nivel de atención utilizado. Combinando los estimativos de costo y utilización, el costo esperado de tratamiento por paciente-año es de $1,028, mientras que el costo promedio durante su expectativa de vida fue de $11,619 por paciente. Los pacientes crónicos con la enfermedad de Chagas tienen un acceso limitado a los cuidados de salud, con un estimativo del 22% que nunca acceden a los servicios de salud.

Conclusión: La enfermedad de Chagas es una condición prevenible que afecta principalmente poblaciones de escasos recursos que habitan en áreas rurales. El costo promedio de encuestar las viviendas para infestación y el rociamiento de las casas infestadas fue menor en comparación a otros estudios, y en relación con los costos de tratamiento. El comportamiento de búsqueda de cuidados de salud y el tipo de afiliación a seguros médicos parecen jugar un papel importante en el tipo de cuidados que reciben los pacientes, generándose algunas dudas

en cuanto al acceso equitativo a los mismos. La prevención de la enfermedad de Chagas en Colombia puede ser costo efectiva y podría contribuir a prevenir inequidades en la salud y en los servicios de atención médica.

Resumen del autor

La enfermedad de Chagas es una de las enfermedades transmitidas por vectores más importantes en América Latina. Muchos pacientes con Chagas pasan años sin ser diagnosticados y debido a que los síntomas de la condición crónica son similares a aquellos de otras afecciones cardiacas, el impacto de la enfermedad no resulta evidente. Esto lleva a subestimar la carga que la enfermedad de Chagas tiene sobre los recursos de salud, lo que además se traduce en una priorización inadecuada de su prevención. Este estudio evalúa los costos de la enfermedad de Chagas en Colombia en relación con los costos de su prevención. Determinar los costos de la enfermedad de Chagas es un desafió. En este estudio usamos una metodología que combina una revisión retrospectiva de costos de tratamiento de pacientes crónicos de Chagas y actividades de control, a la vez que estimamos la utilización de servicios de salud a través de consensos de expertos. Los resultados muestran que los costos de tratamiento de pacientes crónicos son sustanciales, aun cuando muchos pacientes no reciben cuidados apropiados. Por otra parte, se muestra que la prevención de la transmisión de la enfermedad de Chagas a través de rociamiento con insecticida, es asequible en Colombia y por lo tanto debería ser realizada en forma sistemática. Este estudio provee los insumos básicos para conducir un estudio integral de evaluación económica de la prevención de la enfermedad de Chagas.
